# Liver transcriptomics-metabolomics integration reveals biological pathways associated with fetal programming in beef cattle

**DOI:** 10.1038/s41598-024-78965-4

**Published:** 2024-11-12

**Authors:** Guilherme Henrique Gebim Polizel, Simara Larissa Fanalli, Wellison J. S. Diniz, Aline Silva Mello Cesar, Nara Regina Brandão Cônsolo, Heidge Fukumasu, Angela Cánovas, Arícia Christofaro Fernandes, Barbara Carolina Teixeira Prati, Édison Furlan, Gabriela do Vale Pombo, Miguel Henrique de Almeida Santana

**Affiliations:** 1 Department of Animal Science, Faculty of Animal Science and Food Engineering, University of São Paulo, Av. Duque de Caxias Norte, 225, Pirassununga, 13635-900 SP Brazil; 2 Department of Animal Sciences, College of Agriculture, Auburn University, Auburn, AL 36849 USA; 3 Department of Food Science and Technology, Luiz de Queiroz College of Agriculture, University of São Paulo, Av. Pádua Dias 11, Piracicaba, 13418-900 SP Brazil; 4 Department of Nutrition and Animal Production, Faculty of Veterinary Medicine and Animal Science, University of São Paulo, Av. Duque de Caxias Norte, 255, 13635- 900 Pirassununga, SP Brazil; 5 Department of Veterinary Medicine, Faculty of Animal Science and Food Engineering, University of São Paulo, Av. Duque de Caxias Norte, 225, Pirassununga, 13635-900 SP Brazil; 6 Department of Animal Biosciences, University of Guelph, 50 Stone Road East, Guelph, ON Canada

**Keywords:** Maternal nutrition, Metabolites, RNA-seq, Systems biology, WGCNA, Animal biotechnology, Functional genomics, Metabolomics

## Abstract

We investigated the long-term effects of prenatal nutrition on pre-slaughter Nelore bulls using integrative transcriptome and metabolome analyses of liver tissue. Three prenatal nutritional treatments were administered to 126 cows: NP (control, mineral supplementation only), PP (protein-energy supplementation in the third trimester), and FP (protein-energy supplementation throughout pregnancy). Liver samples from 22.5 ± 1-month-old bulls underwent RNA-Seq and targeted metabolomics. Weighted correlation network analysis (WGCNA) identified treatment-associated gene and metabolite co-expression modules, further analyzed using MetaboAnalyst 6.0 (metabolite over-representation analysis and transcriptome-metabolome integrative analysis) and Enrichr (gene over-representation analysis). We identified several significant gene and metabolite modules, as well as hub components associated with energy, protein and oxidative metabolism, regulatory mechanisms, epigenetics, and immune function. The NP transcriptome-metabolome analysis identified key pathways (aminoacyl t-RNA biosynthesis, gluconeogenesis, and PPAR signaling) and hub components (glutamic acid, SLC6A14). PP highlighted pathways (arginine and proline metabolism, TGF-beta signaling, glyoxylate and dicarboxylate metabolism) with arginine and ODC1 as hub components. This study highlights the significant impact of prenatal nutrition on the liver tissue of Nelore bulls, shedding light on critical metabolic pathways and hub components related to energy and protein metabolism, as well as immune system and epigenetics.

**Supplementary Information:**

The online version contains supplementary material available at 10.1038/s41598-024-78965-4.

## Introduction

Most fetal programming studies assessing the effects of prenatal nutrition on the liver link it to clinical diseases, such as non-alcoholic fatty liver disease^1–3^. The liver, the central organ of metabolism, fulfills several vital functions. Beyond its pivotal role as the primary site for energy metabolism (lipids and carbohydrates), it intricately processes bilirubin, bile acids, xenobiotics, facilitates protein synthesis, and contributes significantly to immune function^4^. However, the liver’s significance in beef cattle production extends to various crucial phenotypes (e.g., feed efficiency)^5,6^, which can be influenced by external factors during the gestational period and have long-term effects on the offspring. Throughout the progeny intrauterine development, maternal nutrition influences epigenetic factors that, in turn, may affect gene expression^7^, ultimately resulting in changes to the metabolic abundance and phenotype.

Studies have consistently highlighted the significant impact of maternal nutrient restriction on fetal development, particularly focusing on the liver. Prezotto et al.^8^ observed an increase in fetal liver weight in nutrient-restricted and subsequently re-alimented beef cows compared to the control group. Similarly, maternal nutrient restriction in ewes impacted the morphology, gene expression, and lipid metabolism of the fetal liver^9^. In our previous study^10^, we identified that including energy-protein supplementation during either the last trimester of pregnancy or the entire gestation period had long-term effects on the offspring. These prenatal supplementation strategies influenced the abundance of specific metabolites, including glycine, hydroxytetradecadienylcarnitine, aminoadipic acid, and carnosine, primarily affecting the oxidative metabolism of the offspring.

Utilizing techniques such as RNA sequencing (RNA-seq; transcriptomics) and mass spectrometry (metabolomics) is important for comprehending the non-linear effects associated with fetal programming in beef cattle. While transcriptomics focuses on gene expression by analyzing mRNA levels, providing insights into which genes are active in specific biological contexts^11^, metabolomics measures the end products of cellular processes, including metabolites involved in metabolic pathways^12^. By integrating these two datasets, researchers can gain a holistic view of how gene expression influences metabolic outcomes, offering deeper insights into the underlying biological mechanisms. The advancement of these technologies and their falling costs^13^ have led to a notable rise in the amount of data generated and the emergence of integrative studies aiming for new biological insights.

Despite facing challenges, such as normalization and processing data from diverse omics platforms, standardizing variables, and reducing data dimensionality, bioinformatics has played a pivotal role in advancing tools for multiomics integration^14^. Thanks to these strides in bioinformatics, researchers can now conduct multi-omics studies, generating a variety of omics data types (e.g., transcriptomics, metabolomics) within the same research project across various fields, such as nutritional genomics and fetal programming. This progress is fueled by the pursuit of a more holistic and comprehensive understanding of biological systems^15^.

**Fig. 1:**
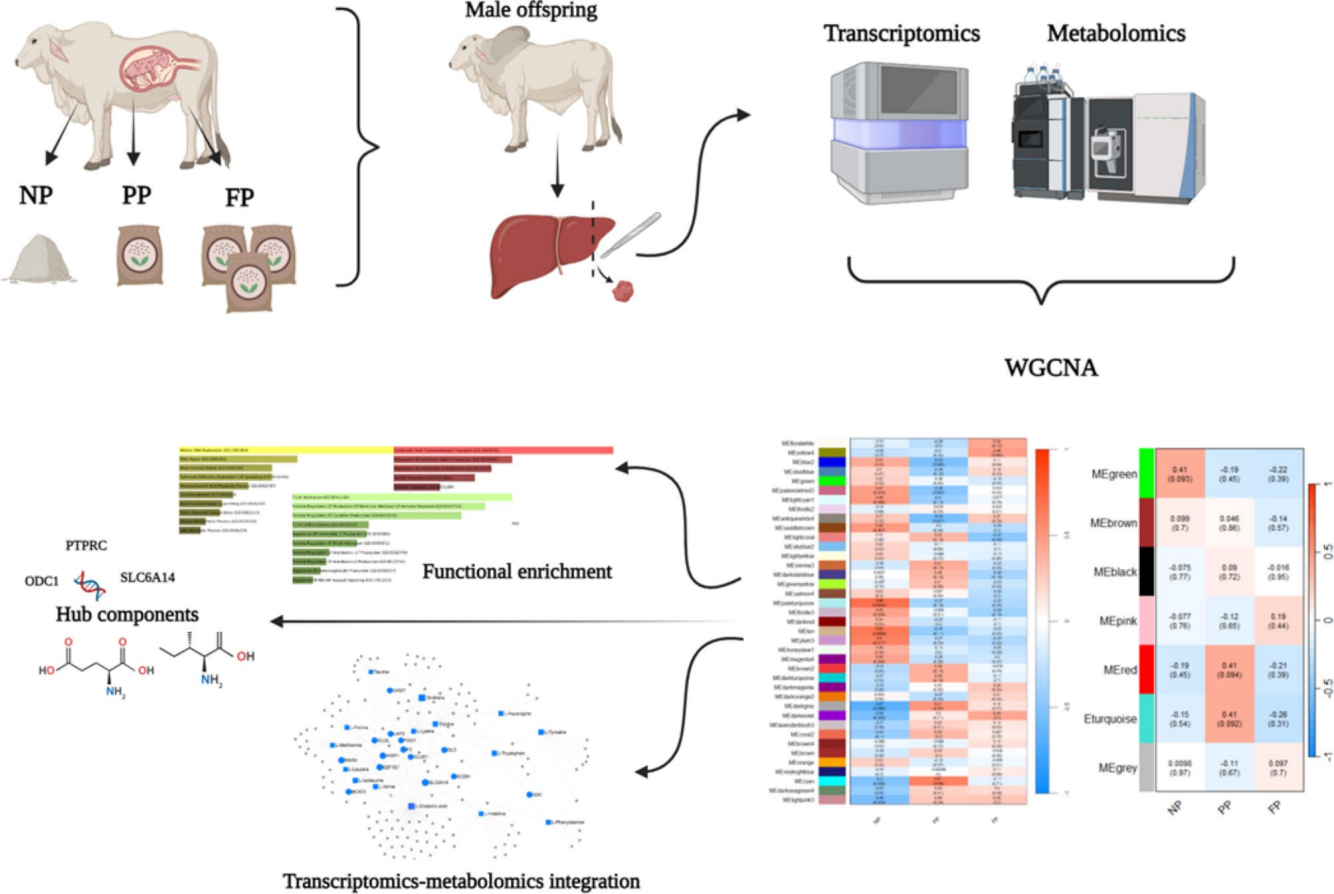
Graphical abstract illustrating the experimental design and main analyses conducted.

The hypothesis of this study is that different prenatal nutritional strategies have long-term effects impacting the liver tissue transcriptome-metabolome through changes in biological pathways. The objectives of the present study were threefold: (1) To evaluate whether the different prenatal nutritional strategies impacted the liver transcriptome and metabolome co-expression networks of bulls in the finishing phase; (2) To evaluate the association between significant co-expressed genes and metabolites and their involvement in biological processes; and (3) To perform an integrated analysis of the liver transcriptome and metabolome to assess the impact of prenatal nutrition on metabolic pathways associated to both omics. Figure 1 provides a visual overview of the main objectives of the study.

## Methods

### Experimental design

All experimental protocols were approved by the Faculty of Animal Science and Food Enginnering (FZEA-USP) committee under the project reference number 1,843,241,117. The experimental protocols were conducted in accordance with the relevant guidelines and regulations. The study design and analysis conform to the ARRIVE recommendations for animal research (https://arriveguidelines.org). All animals used in this experiment were provided by the campus of the FZEA-USP.

In this study, 126 Nelore cows (3.52 ± 1.43 years of age) and their offspring were included to investigate the effects of different prenatal treatments. The cows were artificially inseminated using semen from four sires, and their pregnancy was confirmed 30 days later. To ensure a balanced experimental design, the dams were divided into three groups of 42 animals based on age, body weight (BW), and body condition score. These groups were then kept in pasture paddocks of *Urochloa brizantha* cv. Marandu equipped with a trough for feed supplement and water. The prenatal treatments administered to the cows were as follows: (1) NP (control) - Not Programmed; (2) PP - Partial Programming; and (3) FP - Full Programming. The NP cows received only mineral supplements throughout pregnancy, which accounted for 0.03% of their BW per day. The PP group received protein-energy supplementation in the third trimester, which accounted for 0.3% of their BW per day. The FP group, on the other hand, received the same protein-energy supplementation from pregnancy confirmation until delivery.

All three groups also received mineral supplementation, which accounted for 0.03% of their BW per day. This mineral supplementation was already included in the protein-energy supplement formulation (as demonstrated in Table 1). The nutritional values of the paddocks, which consisted of *Urochloa brizantha* cv. Marandu, were similar across the groups during pregnancy. Briefly, the TDN (Total Digestible Nutrients) values were 63.07% for the NP group, 64.1% for the PP group, and 61.43% for the FP group. The CP (Crude Protein) values were 7.38% for the NP group, 7.82% for the PP group, and 7.40% for the FP group. Finally, the NDF (Neutral Detergent Fiber) values were 59.03% for the NP group, 61.43% for the PP group, and 58.49% for the FP group. Further information regarding the pasture conditions, as well as the phenotypic and metabolic impacts of the treatments (NP, PP, and FP) on dams, were more thoroughly evaluated in the study published elsewhere^16^.

**Table 1:** Composition and nutritional content of the maternal supplement.

Ingredients	Mineral supplement	Protein-energy supplement
Corn (%) Soybean meal (%) Dicalcium phosphate (%) Urea 45% (%) Salt (%) Minerthal 160 MD (%)* Total digestible nutrients (%) Crude protein (%) Non-protein nitrogen (%) Acid detergent fiber (%) Neutral detergent fiber (%) Fat (%) Calcium (g/kg) Phosphorus (g/kg)	35.00 - 10.00 - 30.00 25.00 26.76 2.79 - 1.25 4.29 1.26 74.11 59.38	60.00 30.00 - 2.50 5.00 2.50 67.55 24.78 7.03 4.76 11.24 2.61 6.20 7.24

*Mineral premix composition (Minerthal company): Calcium = 8.6 g/kg; Cobalt = 6.4 mg/kg; Copper = 108 mg/kg; Sulfur = 2.4 g/kg; Fluorine = 64 mg/kg; Phosphorus = 6.4 g/kg; Iodine = 5.4 mg/kg; Manganese = 108 mg/kg; Selenium = 3.2 mg/kg; Zinc = 324 mg/kg; Sodium monensin = 160 mg/kg^86^.

Following calving, protein-energy supplementation was discontinued, and all offspring, irrespective of their prenatal nutritional treatment, were subjected to identical health protocols and nutritional regimens, remaining in common groups until weaning at 240 ± 28 days. Throughout this period, cows received the same mineral supplementation (0.03% of BW) as during the pregnancy phase and were maintained within an extensive pasture system consisting of *Urochloa brizantha* cv. Marandu paddocks.

### Post-weaning and finishing managements

Post-weaning, animals were separated by sex, regardless of their prior treatment, and were reared until the end of the developmental phase at 570 ± 28 days under the same nutritional management. During this phase, young bulls were provided with two distinct supplements: an energetic supplement (TDN = 67.55%; CP = 24.78%; NDF = 11.24%; Fat = 2.61%; 0.3% of BW) during the dry season (winter), and a protein supplement (TDN = 53.15%; CP = 30.03%; NDF = 9.14%; Fat = 1.65%; 0.1% of BW) throughout the wet season (summer). From calving until 570 ± 28 days of age, the young bulls grazed on *Urochloa brizantha* cv. Marandu pastures with free access to water.

The start of the finishing phase for the 63 bulls (NP = 22 bulls, PP = 20 bulls, and FP = 21 bulls) occurred at 570 ± 28 days of age, reaching their slaughter at 676 ± 28 days. Throughout this phase, the bulls were provided with three distinct diets: an adaptation diet for the initial 15 days, characterized by Dry Matter (DM) content of 48.1%, TDN of 71.0%, CP of 15.0%, NDF of 36.5%, Fat content of 3.2%, and Dry Matter Intake (DMI) at 2.21% of BW; a subsequent diet for 35 days (DM = 53.6%; TDN = 73.6%; CP = 14.0%; NDF = 31.1%; Fat = 3.4%; DMI = 2.20% of BW); and a final diet for 56 days (DM = 60.6%; TDN = 76.2%; CP = 13.0%; NDF = 25.8%; Fat = 3.7%; DMI = 2.04% of BW). Upon completion of the finishing phase, the animals were slaughtered at the FZEA/USP school slaughterhouse, situated approximately 500 m from the feedlot facilities. The slaughter and subsequent carcass processing strictly met the guidelines stipulated by the Ministry of Agriculture, Livestock, and Supply of Brazil (MAPA), as outlined in the Normative Instruction No. 9 of 2004. To further contextualize this study, a summary table (Table 2) of key phenotypic effects evaluated during the finishing phase and published in other studies^10,17^ is provided below; all phenotypes presented were collected at slaughter.

**Table 2:** Main phenotypic effects of prenatal nutrition on bulls in the finishing phase.

Traits	NP	PP	FP	*p* value
LW (kg)	7.227 ± 0.734	7.295 ± 0.779	7.286 ± 0.714	0.92
BW (kg)	591.2 ± 40.05	602.6 ± 49.65	597.4 ± 51.06	0.68
HCW (kg)	348.1 ± 4.61	352.7 ± 4.70	356.1 ± 4.73	0.61
CCW (kg)	344.2 ± 6.12	349.3 ± 4.79	353.9 ± 4.81	0.58
REA (cm^2^)	97.6 ± 1.05	98.2 ± 0.96	97.4 ± 0.92	0.70
SFT (mm)	7.81 ± 0.28	8.21 ± 0.33	8.69 ± 0.39	0.08

LW – liver weight; BW – body weight; HCW – hot carcass weight; CCW – cold carcass weight; REA – ribeye area; SFT – subcutaneous fat thickness. Data presented in this table were previously published in Polizel et al.^10^ and Fernandes et al.^17^.

### Liver tissue sample collection

Following slaughter, all bulls had their liver sampled for subsequent metabolomics and transcriptomics analyses. Samples were collected immediately post-slaughter (within 15 min), rapidly snap-frozen in liquid nitrogen, and subsequently stored in an ultrafreezer at -80 °C until further processing. This protocol was consistently followed for all samples, minimizing the potential for mRNA degradation due to prolonged intervals between slaughter and sampling. The liver tissue samples were precisely obtained from the distal portion of the left lobe. From the initial cohort (*n* = 63), 15 male offspring were selected for transcriptomics analysis, comprising 5 randomly chosen animals from each nutritional treatment group. Similarly, 18 animals were selected for liver tissue metabolomics analysis, including the same 15 individuals from the transcriptomics subset, with an additional sample from each treatment group. All the selected animals had the same sire.

### RNA extraction, processing, and sequencing

RNA extraction was performed using the TRIzol reagent (Life Technologies, Carlsbad, CA, USA), following the manufacturer’s instructions. Total RNA was extracted from 100 mg of liver tissue, quantified using the DS-11 spectrophotometer (Denovix, Wilmington, DE, USA), and assessed for RNA integrity using the Bioanalyzer 2100 (Agilent, Santa Clara, CA, USA). The average RNA integrity number (RIN) across the samples was 7.1 (NP = 7.0 ± 0.1; PP = 7.1 ± 0.1; and FP = 7.0 ± 0.1).

For library preparation, 0.1–1 µg of RNA was used following the protocols outlined in the TruSeq Stranded mRNA Reference Guide (Illumina, San Diego, CA, USA). Quantification of the libraries was performed via quantitative PCR (35 cycles of 95 °C–30 s) utilizing the KAPA Library Quantification kit (KAPA Biosystems, Foster City, CA, USA), and the average library size was evaluated using the Bioanalyzer 2100 (Agilent, Santa Clara, CA, USA). The clustering and sequencing of the 15 samples were performed on a single flow-cell sequencing lane using the TruSeq PE Cluster kit v3-cBot-HS (Illumina, San Diego, CA, USA) based on a paired-end approach. The sequencing was performed on the HiSeq2500 platform (Illumina, San Diego, CA, USA), utilizing the TruSeq Stranded mRNA kit, following the manufacturer’s instructions. The sequencing analysis was carried out by the NGS Soluções Genômicas company (Piracicaba, São Paulo, Brazil).

### Metabolomic sample processing and targeted metabolomics

Liver tissue metabolite extraction was conducted using a solvent blend comprising 85 mL of ethanol high-performance liquid chromatography (HPLC) grade and 15 mL of phosphate buffer (0.01 M, pH = 7.5 at 25 °C). All procedures were meticulously executed below 0 °C, with dry ice used to prevent any degradation processes. Subsequently, the samples were weighed and homogenized using a bead-based homogenizer (20 s at 5500 RPM) with the aforementioned extraction solvent. This homogenization procedure was repeated three times, followed by centrifugation (10,000 x g for 5 min). The resulting supernatant was then carefully transferred to tubes and quickly stored in an ultra-cold freezer until metabolite quantification using the AbsoluteIDQ p180 Kit. Further details on the extraction protocol can be found in Zukunft et al.^18^.

Apex Science company (Campinas, São Paulo, Brazil) performed the metabolomics analysis using the AbsoluteIDQ p180 Kit by Biocrates Life Sciences AG, covering 188 metabolites. The analysis covers various metabolite classes, including amino acids, biogenic amines, acylcarnitines, lysophosphatidylcholines, phosphatidylcholines, sphingolipids, and hexose. Amino acids and biogenic amines were analyzed using liquid chromatography tandem-mass spectrometry (HPLC-MS/MS). In contrast, other metabolites were analyzed using flow injection analysis-tandem mass spectrometry (FIA-MS/MS). MetIDQ software was used for data analysis, and metabolite concentrations were calculated using internal standards. Biocrates establishes metabolite-specific limits of detection (LOD) experimentally. More details are available in Polizel et al.^10^.

### Transcriptomics data filtering, alignment, data transformation, and normalization

Initially, the quality analysis of the raw RNA-seq data was performed using the FASTQC program version 0.11.9 (http://www.bioinformatics.babraham.ac.uk/projects/fastqc/). The data was then submitted to filtering to remove adapter sequences and low-complexity reads using SeqyClean version 1.9.10 ^19^. The clean reads were aligned to the *Bos taurus* ARS-UCD1.2.110 reference genome (available at: https://ftp.ensembl.org/pub/release-110/fasta/bos_taurus/) using the STAR aligner v. 020201 ^20^. The precise numbers regarding initial filtering and aligned reads are available in Additional file 1.

After filtering out genes with zero counts (non expressed), with low expression (less than one count per million per sample on average), and those with fewer than ten counts in at least three samples, the count per million table was log-transformed. The data was further adjusted for age effect using the “*removeBatchEffect*” function from the limma R-package. Ultimately, after final filtering and normalization, the dataset comprised 13,621 genes.

### Metabolomics data filtering, scaling, and normalization

Identified metabolites exhibiting over 70% of samples below or above the LOD or with uniform values across samples were excluded from the dataset, resulting in 180 remaining metabolites. For the metabolites retained post-filtering, LOD values were substituted with the minimum value if below the LOD and with the maximum value if above the LOD for each respective variable. Then, the data was adjusted for age effect using the function “*removeBatchEffect*” from the limma R-package. The resulting dataset was autoscaled to meet the normalization parameters.

### WGCNA analysis

We used the Weighted Gene Co-expression Network Analysis (WGCNA) R-package v. 1.72–5^21^ to investigate the co-expression pattern of genes and metabolites in response to the effects of prenatal nutrition. Furthermore, we investigated hub genes and hub metabolites within the networks that were potentially the main drivers of liver function.

The initial stage of the WGCNA analysis involved converting our nutritional treatment groups (NP, PP, and FP) into binary variables via dummy transformation to comply with the WGCNA workflow. Using the WGCNA framework, correlation coefficients between gene pairs and metabolite pairs were computed separately to create the adjacency matrices. Soft thresholds for transcriptomics (power = 12, R^2^ = 0.83) and metabolomics (power = 15, R^2^ = 0.50) were then selected to construct scale-free co-expression networks. As the number of metabolites is limited, the scale-free topology fit index did not reach values exceeding 0.8 for a suitable power level^21^. Because this dataset does not meet the assumptions of a scale-free network, we assessed the mean connectivity, as recommended by^22^. Following the protocol outlined for proteome and metabolome datasets^23^, we chose a soft threshold of 15, resulting in a mean connectivity of 8.40.

Subsequently, the adjacency matrix was transformed into a topological overlap matrix. The cluster analysis was then carried out to detect modules, ensuring a minimum of 30 genes and five metabolites per module for transcriptomics and metabolomics, respectively. Modules with a correlation value (r) ≥ 0.75 were merged within each dataset. Through hierarchical clustering, genes or metabolites with a similar expression/abundance pattern across samples were grouped into the same module and arbitrarily labeled by color. The modules were then summarized based on the eigengene concept, and the module eigengene values were correlated to the groups (NP, PP, and FP). Modules were considered significant when the p-value was ≤ 0.1, according to WGCNA manual guidelines. Heatmaps were then generated to represent significant correlations between the groups and the genes and metabolite modules. Identification of core genes and metabolites within significant modules was carried out using the “*chooseTopHubInEachModule*” function implemented in the WGCNA package.

### Functional enrichment analysis

Functional enrichment analysis to uncover associated biological processes was conducted for both genes and metabolites within each significant module. Gene functional enrichment analysis was performed using Enrichr (https://maayanlab.cloud/Enrichr/) to identify over-represented Gene Ontology (GO) biological processes. Metabolite enrichment analysis for significant modules was conducted by MetaboAnalyst v. 6.0 (https://www.metaboanalyst.ca/) using the function “Over Representation Analysis” and based on the Kyoto Encyclopedia of Genes and Genomes (KEGG). Biological processes and metabolic pathways were considered significant when the adjusted p-value was ≤ 0.1.

### Data integration

For data integration, we conducted a “Joint Pathway Analysis” and a “Network Analysis” using MetaboAnalyst 6.0 (https://www.metaboanalyst.ca/). Our inputs comprised all genes and metabolites from significantly associated modules. The “Joint Pathway Analysis” allowed us to identify pathways that underlie both metabolites and genes associated with each prenatal nutritional treatment (NP, PP, and FP). Each treatment was individually analyzed to identify the biological pathways affected differentially according to the prenatal nutritional strategy. This analysis method relies on tight integration, where genes and metabolites are combined into a single query for enrichment analysis (KEGG pathway). Biological pathways with adjusted p-value ≤ 0.1 were considered affected by the prenatal diet. The “Network Analysis” was used to explore and visualize the gene-metabolite interaction network based on the STITCH database (http://stitch.embl.de/). This analysis enabled us to identify the genes and metabolites with the highest network connectivity, according to their connectivity degree and betweenness centrality measures. The gene-metabolite interaction network allows for the exploration and visualization of interactions between functionally related metabolites and genes. Associations between metabolites and genes were sourced from STITCH, focusing exclusively on highly confident interactions. Most connections in STITCH are derived from co-mentions in PubMed abstracts, highlighting reactions involving similar chemical structures and molecular activities.

## Results

### WGCNA analysis

**Fig. 2:**
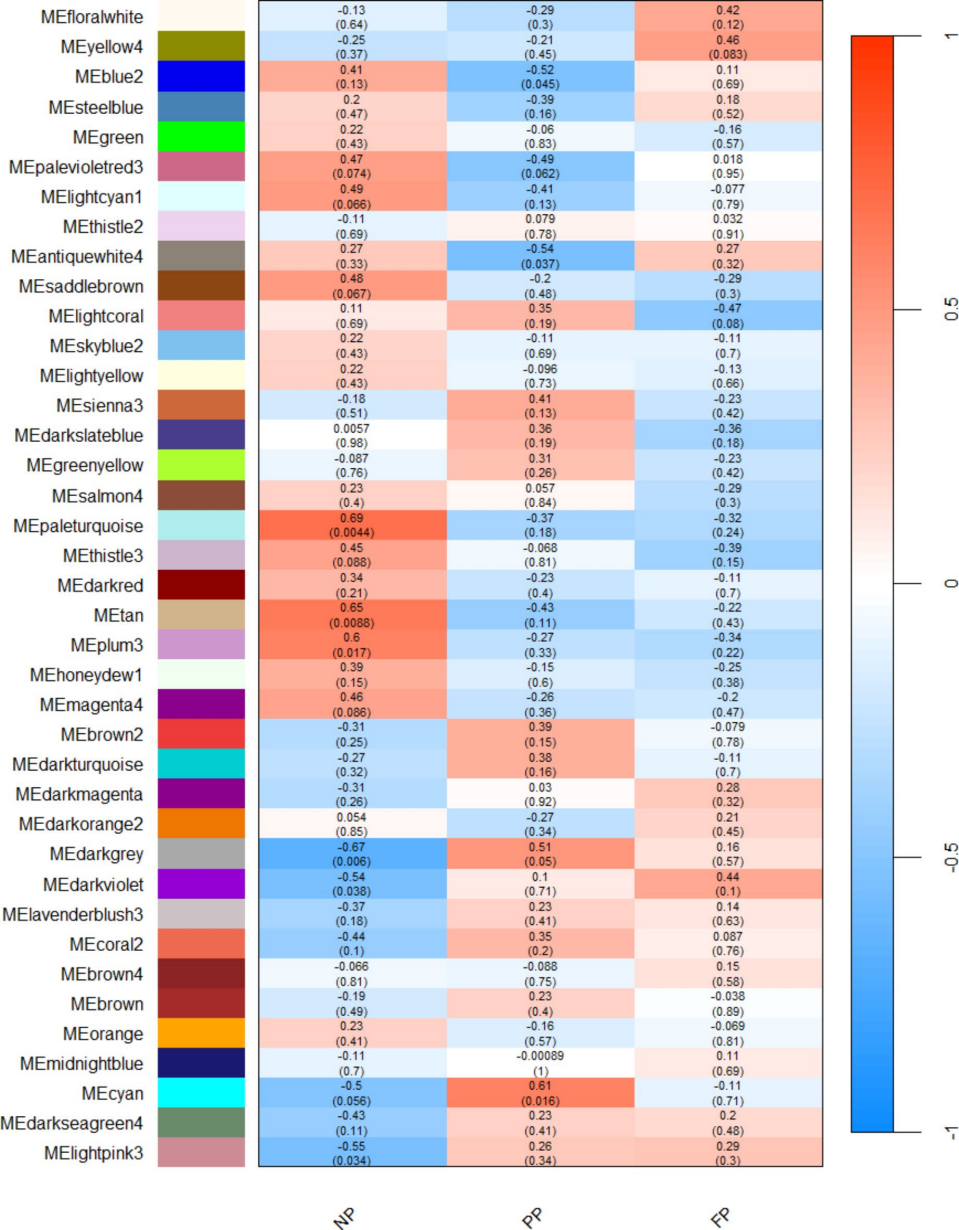
Gene module–treatment correlations heatmap. Each row corresponds to a module, and each column corresponds to a prenatal nutritional treatment group (NP, PP, and FP). Each cell contains the corresponding correlation and p-value. The table is colour-coded by correlation, according to the colour legend.

The gene co-expression analysis resulted in 13 out of 17 significantly associated modules (*p* ≤ 0.1) for the NP group (palevioletred3, lightcyan1, saddlebrown, paleturquoise, thistle3, tan, plum3, magenta4, darkgrey, darkviolet, coral2, cyan and lightpink3), five significant modules for the PP (blue2, palevioletred3, antiquewhite4, darkgrey, and cyan), and three significant modules for the FP group (yellow4, lightcoral and darkviolet). The significant correlations ranged from |0.44| to |0.69| in the NP group modules, |0.49| to |0.61| in the PP group modules, and |0.44| to |0.47| in the FP group modules. As demonstrated in Fig. 2, we identified shared modules between NP and PP groups (palevioletred3, darkgrey, cyan) and between NP and FP groups (darkviolet). Interestingly, all the shared modules exhibited opposite correlation signals between the groups.

**Fig. 3:**
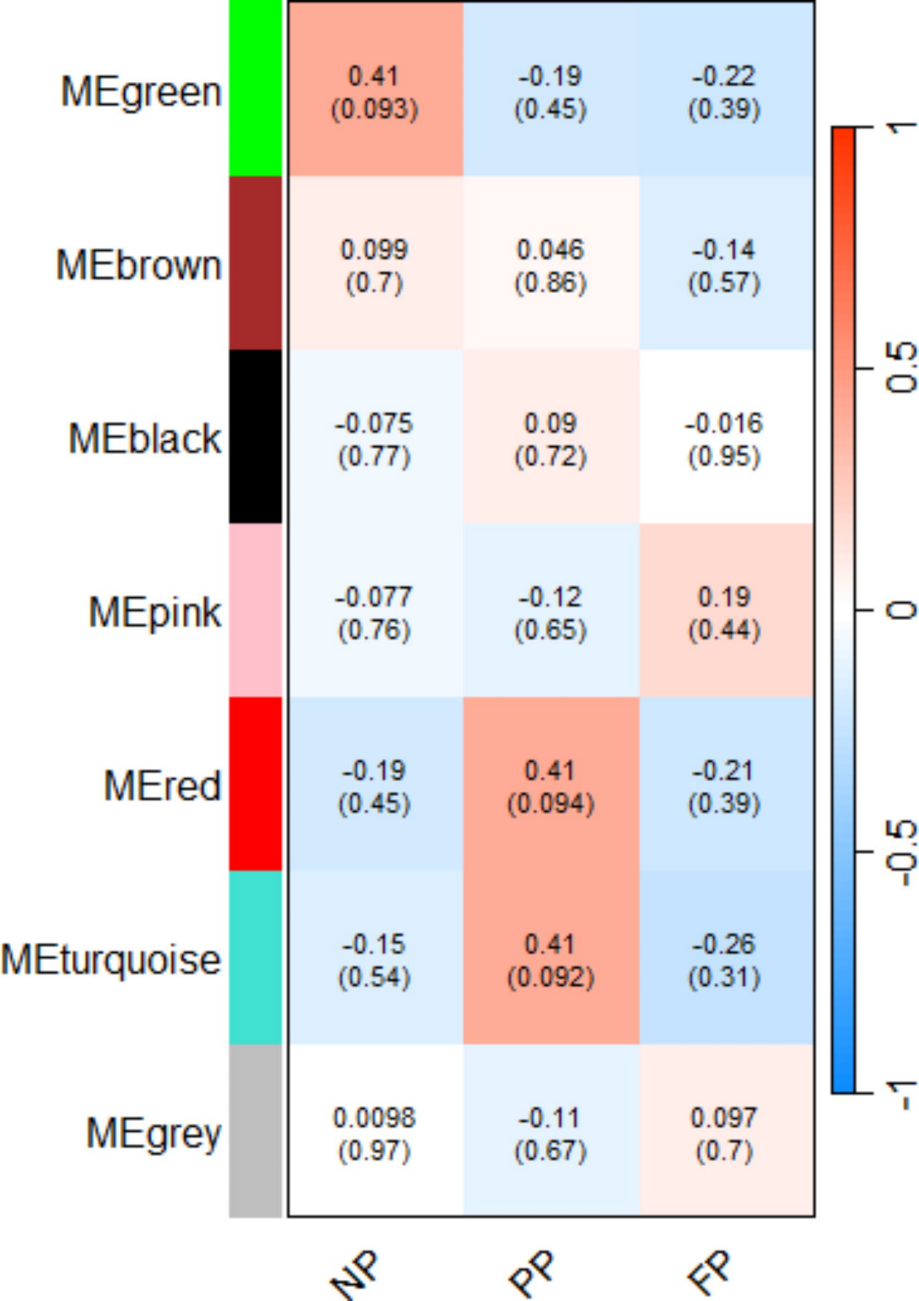
Metabolite module–treatment correlations heatmap. Each row corresponds to a metabolite module, and each column corresponds to a prenatal nutritional treatment group (NP, PP, and FP). Each cell contains the corresponding correlation and p-value. The table is colour-coded by correlation, according to the colour legend.

Regarding to the co-abundant metabolites (Fig. 3), we found one significant module for the NP group (green), two for the PP (red and turquoise) and none for the FP group. The correlation was 0.41 for the NP group and 0.41 for both significant modules in the PP group (Fig. 3).

### Hub genes and hub metabolites

The hub genes (Table 3) linked to the significant modules in the NP group were identified (palevioletred3 = *GSS*; lightcyan1 = *MINK1*; saddlebrown = *MAP4K2*; paleturquoise = *CKAP2*; thistle3 = *HMGCR*; tan = *ZNF311*; plum3 = *EBP*; magenta4 = *ENSBTAG00000053419*; darkgrey = *EIF4G2*; darkviolet = *ENSBTAG00000054363*; coral2 = *ZNF655*; cyan = *ETF1*; and lightpink3 = *RIC8B*), as well as the hub genes associated to the PP group (antiquewhite4 = *SIAE*; blue2 = *ST3GAL5*; cyan = *ETF1*; darkgrey = *EIF4G2*; and palevioletred3 = *GSS*) and FP group (yellow4 = *SLC12A8*; lightcoral = *PTPRC*; and darkviolet = *ENSBTAG00000054363*).

**Table 3:** Hub genes associated to the different nutritional treatment groups (NP, PP, and FP) and their corresponding significant modules in WGCNA analysis.

Treatment	Module	Gene Code	Gene Name
**NP**	palevioletred3	ENSBTAG00000003504	*GSS*
	lightcyan1	ENSBTAG00000004907	*MINK1*
	saddlebrown	ENSBTAG00000001037	*MAP4K2*
	paleturquoise	ENSBTAG00000021162	*CKAP2*
	thistle3	ENSBTAG00000007840	*HMGCR*
	tan	ENSBTAG00000008349	*ZNF311*
	plum3	ENSBTAG00000009287	*EBP*
	magenta4	ENSBTAG00000053419	
	darkgrey	ENSBTAG00000020308	*EIF4G2*
	darkviolet	ENSBTAG00000054363	
	coral2	ENSBTAG00000030951	*ZNF655*
	cyan	ENSBTAG00000011415	*ETF1*
	lightpink3	ENSBTAG00000000147	*RIC8B*
**PP**	antiquewhite4	ENSBTAG00000009412	*SIAE*
	blue2	ENSBTAG00000011601	*ST3GAL5*
	cyan	ENSBTAG00000011415	*ETF1*
	darkgrey	ENSBTAG00000020308	*EIF4G2*
	palevioletred3	ENSBTAG00000003504	*GSS*
**FP**	yellow4	ENSBTAG00000000496	*SLC12A8*
	lightcoral	ENSBTAG00000023144	*PTPRC*
	darkviolet	ENSBTAG00000054363	

The hub metabolite identified in the NP green module was isoleucine. In the PP group, we identified the PC ae C34:2 in the red module and the PC ae C44:5 in the turquoise module (Table 4).

**Table 4:** Hub metabolites associated to the different nutritional treatment groups (NP, PP, and FP) and their corresponding significant modules in WGCNA analysis.

Treatment	Module	Metabolite
NP	green	Isoleucine
PP	red	PC ae C34:2
	turquoise	PC ae C44:5

### Gene and metabolite functional enrichment analysis

Based on the gene functional enrichment analysis of each significant module, we found several important GO biological processes (adj. p-value ≤ 0.1) related to prenatal treatments in bulls. In the NP group (Table 5), we identified eight functionally enriched modules (cyan, darkgrey, lightcyan1, lightpink3, paleturquoise, palevioletred3, plum3 and thistle3), most of them with biological processes related to epigenetic mechanisms and regulatory processes (e.g., Regulation of translation, DNA repair, Positive regulation of transcription from RNA polymerase II promoter in response to stress, etc.).

**Table 5:** Top 10 GO biological processes related to the genes within the significant modules in the NP group.

Module	GO Biological Process	Adjusted *p*-value
Cyan	mRNA Splicing, Via Spliceosome (GO:0000398)	4.8E-13
	Ribosome Biogenesis (GO:0042254)	4.8E-13
	mRNA Processing (GO:0006397)	8.2E-12
	RNA Splicing, Via Transesterification Reactions with Bulged Adenosine as Nucleophile (GO:0000377)	4.3E-11
	Ribonucleoprotein Complex Biogenesis (GO:0022613)	3.7E-9
	Ribosomal Small Subunit Biogenesis (GO:0042274)	1.0E-8
	Regulation of Translation (GO:0006417)	7.7E-8
	Maturation of SSU-rRNA (GO:0030490)	7.7E-8
	rRNA Processing (GO:0006364)	3.8E-7
	RNA Processing (GO:0006396)	1.1E-6
Darkgrey	Response to Endoplasmic Reticulum Stress (GO:0034976)	3.2E-5
	Ribosome Biogenesis (GO:0042254)	2.3E-4
	Golgi Vesicle Transport (GO:0048193)	2.3E-4
	Organelle Organization (GO:0006996)	6.5E-4
	ERAD Pathway (GO:0036503)	6.5E-4
	Regulation of Translation (GO:0006417)	7.6E-4
	Regulation of Translational Initiation (GO:0006446)	9.3E-4
	Regulation of Protein Ubiquitination (GO:0031396)	9.3E-4
	Endoplasmic Reticulum to Golgi Vesicle-Mediated Transport (GO:0006888)	0.001
	Ribonucleoprotein Complex Biogenesis (GO:0022613)	0.001
Lightpink3	Regulation of Immune Response (GO:0050776)	0.065
	Positive Regulation of Transcription From RNA Polymerase II Promoter in Response To Stress (GO:0036003)	0.065
	snRNA Metabolic Process (GO:0016073)	0.066
Paleturquoise	Mitotic Sister Chromatid Segregation (GO:0000070)	1.5E-8
	Microtubule Cytoskeleton Organization Involved in Mitosis (GO:1902850)	1.5E-8
	Mitotic Cytokinesis (GO:0000281)	1.5E-8
	Positive Regulation of Mitotic Sister Chromatid Separation (GO:1901970)	3.1E-8
	Cytoskeleton-Dependent Cytokinesis (GO:0061640)	1.7E-7
	Mitotic Spindle Organization (GO:0007052)	2.2E-7
	Regulation of Cell Cycle Process (GO:0010564)	5.4E-7
	Positive Regulation of Cell Cycle Process (GO:0090068)	3.0E-6
	Sister Chromatid Segregation (GO:0000819)	3.4E-6
	Mitotic Spindle Assembly (GO:0090307)	1.5E-5
Palevioletred3	Mitotic DNA Replication (GO:1902969)	0.008
	DNA Repair (GO:0006281)	0.042
	Base-Excision Repair (GO:0006284)	0.041
	Substrate Adhesion-Dependent Cell Spreading (GO:0034446)	0.041
	Monocarboxylic Acid Metabolic Process (GO:0032787)	0.059
	Gluconeogenesis (GO:0006094)	0.072
	Base-Excision Repair, Gap-Filling (GO:0006287)	0.075
	Store-Operated Calcium Entry (GO:0002115)	0.075
	Hexose Biosynthetic Process (GO:0019319)	0.094
	DNA Metabolic Process (GO:0006259)	0.096
Plum3	Cellular Component Assembly (GO:0022607)	0.057
	Synaptonemal Complex Assembly (GO:0007130)	0.057
	Synaptonemal Complex Organization (GO:0070193)	0.057
	Regulation of Meiotic Cell Cycle (GO:0051445)	0.078
	Anaphase-Promoting Complex-Dependent Catabolic Process (GO:0031145)	0.078
Thistle3	Sterol Biosynthetic Process (GO:0016126)	1.1E-13
	Secondary Alcohol Biosynthetic Process (GO:1902653)	3.2E-12
	Cholesterol Biosynthetic Process (GO:0006695)	3.2E-12
	Cholesterol Metabolic Process (GO:0008203)	5.6E-11
	Steroid Biosynthetic Process (GO:0006694)	1.3E-4
	Sterol Metabolic Process (GO:0016125)	2.2E-4
	Isoprenoid Biosynthetic Process (GO:0008299)	0.002
	Regulation of Amyloid-Beta Clearance (GO:1900221)	0.008
	Lipid Biosynthetic Process (GO:0008610)	0.008
	Negative Regulation of Protein Transport (GO:0051224)	0.020
Lightcyan1	Carboxylic Acid Transmembrane Transport (GO:1905039)	0.096

In the PP group (Table 6) all significant modules were functionally enriched (blue2, palevioletred3, antiquewhite4, darkgrey, and cyan). In addition to the biological processes associated with epigenetic mechanisms, we retrieved significant processes related to energy and protein metabolism (e.g., Unsaturated fatty acid metabolic process, Serine family amino acid metabolic process, Gluconeogenesis, etc.).

**Table 6:** Top 10 GO biological processes related to the genes within the significant modules in the PP group.

Modules	GO Biological Processes	Adjusted *p*-value
Antiquewhite4	Unsaturated Fatty Acid Metabolic Process (GO:0033559)	0.054
Blue2	Endocytosis (GO:0006897)	0.009
	Serine Family Amino Acid Metabolic Process (GO:0009069)	0.067
Cyan	mRNA Splicing, Via Spliceosome (GO:0000398)	4.8E-13
	Ribosome Biogenesis (GO:0042254)	4.8E-13
	mRNA Processing (GO:0006397)	8.2E-12
	RNA Splicing, Via Transesterification Reactions with Bulged Adenosine As Nucleophile (GO:0000377)	4.3E-11
	Ribonucleoprotein Complex Biogenesis (GO:0022613)	3.7E-9
	Ribosomal Small Subunit Biogenesis (GO:0042274)	1.0E-8
	Regulation of Translation (GO:0006417)	7.7E-8
	Maturation of SSU-rRNA (GO:0030490)	7.7E-8
	rRNA Processing (GO:0006364)	3.8E-7
	RNA Processing (GO:0006396)	1.1E-6
Darkgrey	Response To Endoplasmic Reticulum Stress (GO:0034976)	3.2E-5
	Ribosome Biogenesis (GO:0042254)	2.3E-4
	Golgi Vesicle Transport (GO:0048193)	2.3E-4
	Organelle Organization (GO:0006996)	6.5E-4
	ERAD Pathway (GO:0036503)	6.5E-4
	Regulation of Translation (GO:0006417)	7.6E-4
	Regulation of Translational Initiation (GO:0006446)	9.3E-4
	Regulation of Protein Ubiquitination (GO:0031396)	9.3E-4
	Endoplasmic Reticulum to Golgi Vesicle-Mediated Transport (GO:0006888)	0.001
	Ribonucleoprotein Complex Biogenesis (GO:0022613)	0.001
Palevioletred3	Mitotic DNA Replication (GO:1902969)	0.008
	DNA Repair (GO:0006281)	0.041
	Base-Excision Repair (GO:0006284)	0.041
	Substrate Adhesion-Dependent Cell Spreading (GO:0034446)	0.041
	Monocarboxylic Acid Metabolic Process (GO:0032787)	0.059
	Gluconeogenesis (GO:0006094)	0.072
	Base-Excision Repair, Gap-Filling (GO:0006287)	0.075
	Store-Operated Calcium Entry (GO:0002115)	0.075
	Hexose Biosynthetic Process (GO:0019319)	0.094
	DNA Metabolic Process (GO:0006259)	0.096

In the FP group (Table 7), we identified two significant modules functionally enriched (lightcoral and yellow4). Among the significant processes, we emphasize that while there was a process related to regulatory mechanisms (negative regulation of nucleic acid-templated transcription (GO:1903507)), most enriched biological processes were associated with immunological mechanisms.

**Table 7:** Top 10 GO biological processes related to the genes within the significant modules in the FP group.

Modules	GO Biological Processes	Adjusted *p*-value
Lightcoral	T Cell Activation (GO:0042110)	9.3E-6
	Positive Regulation of Production of Molecular Mediator Of Immune Response (GO:0002702)	1.4E-5
	Positive Regulation of Cytokine Production (GO:0001819)	2.6E-5
	T Cell Differentiation (GO:0030217)	8.5E-4
	Regulation of Interleukin-2 Production (GO:0032663)	8.5E-4
	Positive Regulation of B Cell Activation (GO:0050871)	0.001
	Positive Regulation of Interleukin-17 Production (GO:0032740)	0.002
	Positive Regulation of Interleukin-2 Production (GO:0032743)	0.002
	Regulation of Immunoglobulin Production (GO:0002637)	0.003
	Regulation of NIK/NF-kappaB Signaling (GO:1901222)	0.004
Yellow4	Negative Regulation of Nucleic Acid-Templated Transcription (GO:1903507)	0.089
	Regulation of Cardiac Muscle Cell Differentiation (GO:2000725)	0.089
	Ephrin Receptor Signaling Pathway (GO:0048013)	0.089

We highlighted the top 10 biological processes of each module and their respective treatment in tables (Tables 5 and 6, and 7). If enriched, modules gathered fewer than ten significant biological processes, all processes were included in their respective table.

**Fig. 4:**
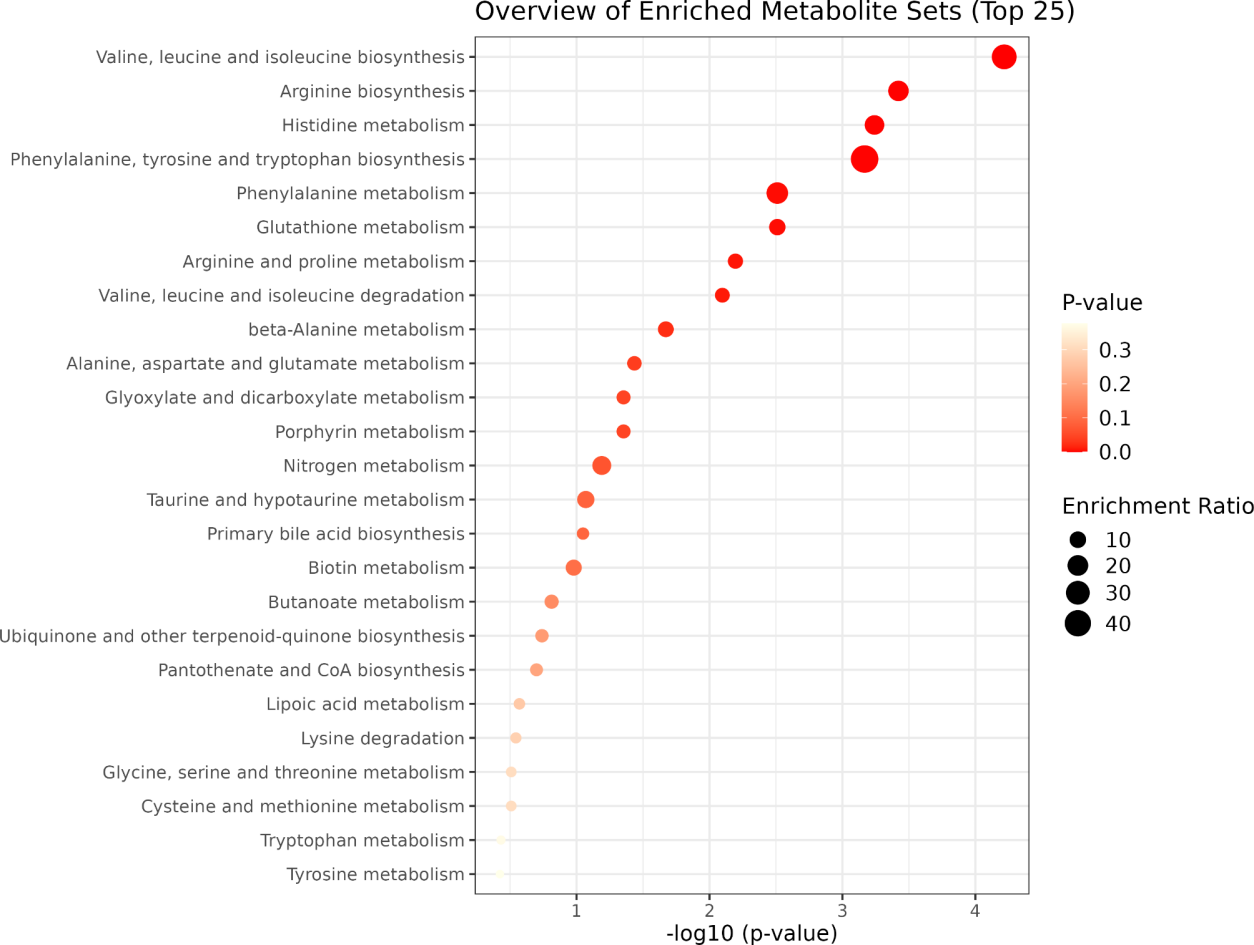
Functional enrichment analysis of metabolites associated with the significant NP group module.

**Fig. 5:**
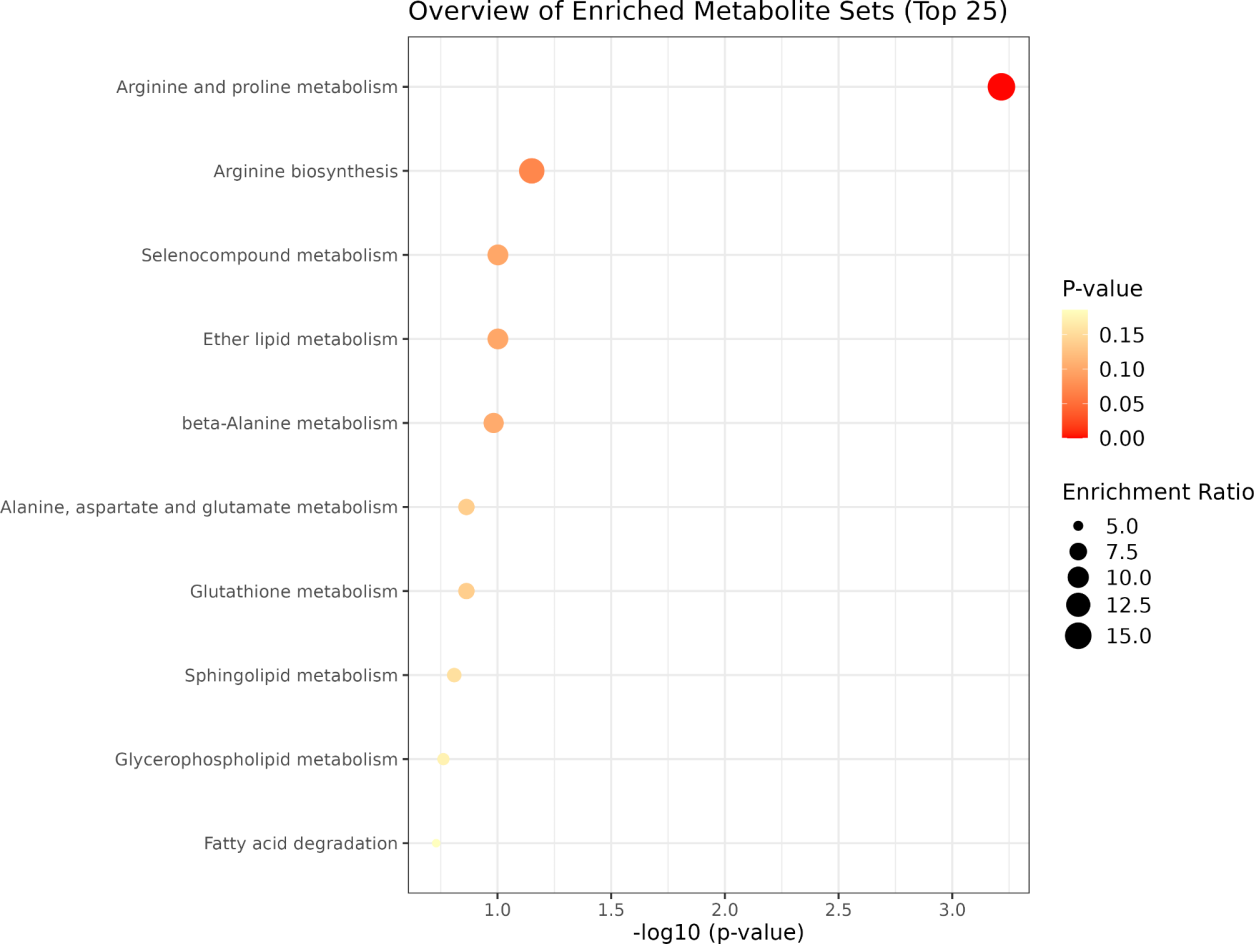
Functional enrichment analysis of metabolites associated with the significant PP group modules.

In the metabolite functional enrichment analysis, we found eight significant biological pathways (Fig. 4) associated with the green module from the NP group (Valine, Leucine, and Isoleucine biosynthesis [*p* = 0.004]; Arginine Biosynthesis [*p* = 0.013]; Histidine metabolism [*p* = 0.013]; Phenylalanine, tyrosine and tryptophan biosynthesis [*p* = 0.013]; Phenylalanine metabolism [*p* = 0.041]; Glutathione metabolism [*p* = 0.041]; Arginine and Proline metabolism [*p* = 0.073]; and Valine, Leucine and Isoleucine degradation [*p* = 0.080]). Regarding the PP group, just one biological pathway (Fig. 5) associated with the turquoise module was identified (Arginine and Proline metabolism [*p* = 0.048]). The red module did not show any significantly enriched pathways associated with the metabolites (*p* > 0.1).

### Transcriptomics-metabolomics integration

Based on the Joint Pathway Analysis performed, we refined the initial results (more than 70 significant pathways for the NP group and more than 50 significant pathways for the PP group) by focusing on the unique pathways associated with each nutritional treatment group (NP and PP). Thus, we identified 19 distinct biological pathways for the NP treatment (*p* ≤ 0.1) associated with several important mechanisms related to energy, protein and vitamin metabolism, epigenetic mechanisms, and systemic cell metabolism. In the PP group, we identified three exclusive biological pathways associated mainly with energy and protein metabolism and cellular processes. The exclusive biological pathways underlying each prenatal nutritional strategy are detailed in Table 8.

**Table 8:** Exclusive biological pathways involved in each prenatal nutritional treatment group (NP and PP) from the transcriptomics-metabolomics integration analysis.

Treatment	Biological Pathway	FDR
NP	Aminoacyl-tRNA biosynthesis	0.024155
	Glycolysis or Gluconeogenesis	0.025383
	Retinol metabolism	0.025738
	Nitrogen metabolism	0.025738
	PPAR signaling pathway	0.034724
	Hippo signaling pathway - multiple species	0.047451
	NF-kappa B signaling pathway	0.047451
	SNARE interactions in vesicular transport	0.047451
	Nicotinate and nicotinamide metabolism	0.051018
	PI3K-Akt signaling pathway	0.058405
	Hippo signaling pathway	0.066801
	Notch signaling pathway	0.066801
	Histidine metabolism	0.066853
	p53 signaling pathway	0.071066
	Cholesterol metabolism	0.077012
	Platelet activation	0.082731
	NOD-like receptor signaling pathway	0.088059
	Th17 cell differentiation	0.088059
	Glycosaminoglycan biosynthesis - heparan sulfate / heparin	0.097841
PP	TGF-beta signaling pathway	0.025467
	Arginine and proline metabolism	0.064294
	Glyoxylate and dicarboxylate metabolism	0.091628

**Fig. 6:**
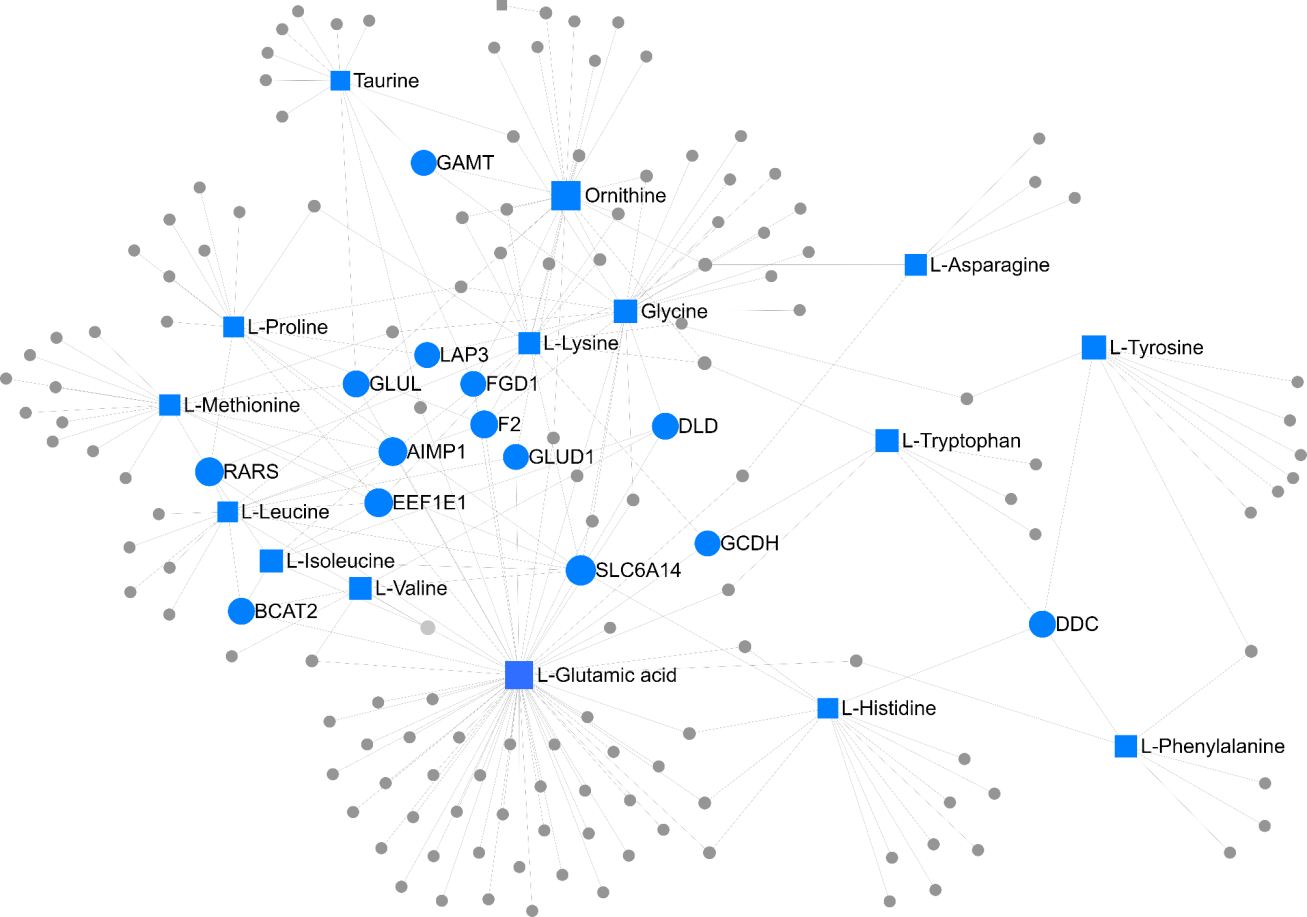
Gene-metabolite network integration of the NP group. Squares represent the metabolites and circles the genes in the network. The top 30 components (selected based on betweenness centrality) are explicitly labeled in the network, whereas the remaining ones are denoted by grey squares or circles.

**Fig. 7:**
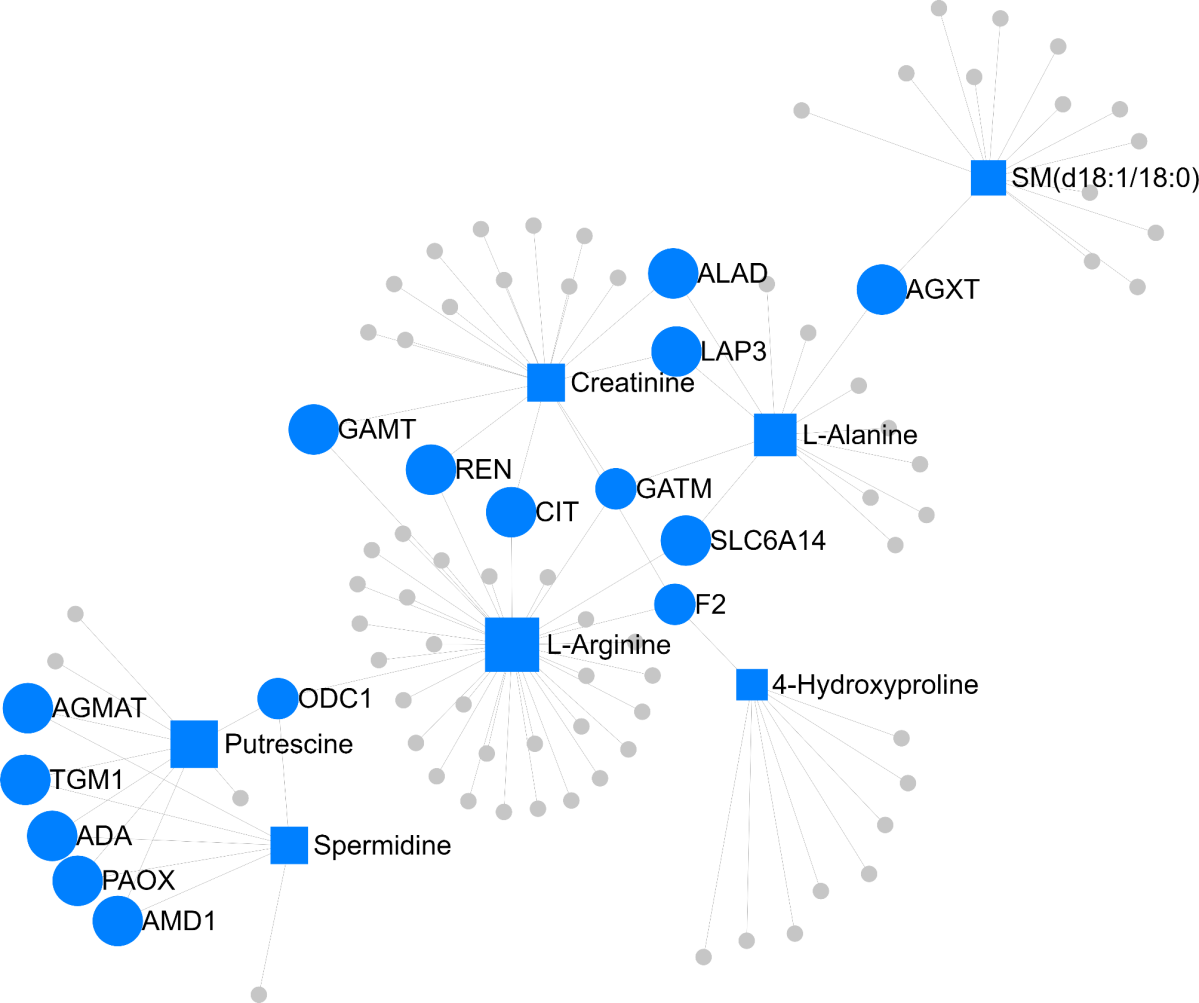
Gene-metabolite network integration of the PP group. Squares represent the metabolites and circles the genes in the network. The top components (selected based on betweenness centrality > 0) are explicitly labeled in the network, whereas the remaining ones are denoted by grey squares or circles.

The gene-metabolite network integration of each nutritional treatment is represented according to Fig. 6 (NP treatment) and Fig. 7 (PP treatment). In the NP group, glutamic acid was the metabolite with the highest connectivity degree and betweenness centrality (degree = 61 and betweenness = 7912.10), and *SLC6A14* was the gene with the greatest network connectivity (degree = 8 and betweenness = 1452.61). Regarding PP group, arginine was the metabolite with the highest network connectivity (degree = 35; betweenness = 3002.80), and *ODC1* was the gene with the highest parameters (degree = 3; betweenness = 903.33).

## Discussion

### NP group is mainly correlated with epigenetic and regulatory mechanisms

The impact of prenatal nutrition on the liver in beef cattle has been studied^24–27^; however, the molecular mechanisms and biological processes involved are still poorly described in the literature.

The co-expression networks were constructed within the sets of liver tissue co-expressed genes and metabolites to uncover the liver transcriptional and metabolic changes involved with fetal programming. Correlations between |0.40| and |0.69| are classified as moderate correlations^28^. Thereby, all the significant modules found (gene expression and metabolite abundance) were considered to be of moderate magnitude (|0.41| ≤ r ≤ |0.69|).

Among the 13 NP group significant gene modules, the paleturquoise and the darkgrey were the most correlated to the NP group (*r* = 0.69 and *r* = -0.67, respectively). The hub gene associated with the paleturquoise module (*CKAP2*) performs functions related to cell division, centrosome function, and mitotic spindle bipolarity, which may impair liver regeneration function^29^. The *CKAP2* gene has also been identified as a hub gene associated with lipid metabolism in chicken liver^30^; however, further studies are required to confirm the association of this gene with lipid metabolism in cattle. The *EIF4G2* (eukaryotic translation initiation factor 4 gamma 2), was identified as a hub gene of the darkgrey module. This gene is a translator activator during cellular stress^31^. Moreover, recent studies have highlighted its significance as a regulator of start codon selectivity^32^. The functions of both hub genes are closely related to the main enriched biological processes, which were associated with the cell cycle, mitosis, and transcriptional and translational regulatory mechanisms. In addition, there are other important biological processes associated with NP group. Lipid metabolism (thistle3 module) and immunological response (lightpink3 module) are important processes in ruminant production. Although we have not performed assessments of feed efficiency and non-esterified fatty acids (NEFA), it is important to note that the liver performs oxidation and metabolic conversion of non-esterified fatty acids, the synthesis of cholesterol and phospholipids, and the formation and secretion of specific classes of lipoproteins^33^. Thus, any impact on fat metabolism could affect several important traits in beef cattle, such as feed efficiency^5^. The effects of maternal nutrition on health parameters are being investigated in some beef cattle studies^34–36^. Prenatal nutritional restriction may weaken the immune system during fetal development and suppress postnatal humoral immune function in beef calves^37^. This may influence the mortality rates^38^, disease incidence^39^ and stress resilience^40^.

In the metabolomics WGCNA, only one module exhibited a significant correlation with the NP group (green). Considering this analysis, isoleucine was identified as the hub metabolite of the module and, consequently, of the NP treatment. Isoleucine is an essential amino acid and one of the three branched-chain amino acids (BCAA) together with leucine and valine. Excessive doses of isoleucine exhibit a potent antagonistic effect on the remaining two BCAAs, leading to a concentration imbalance in plasma and brain amino acid levels that contributes to decreased feed consumption and compromised weight gain and feed efficiency^41^. Moreover, the effects of reduced dietary isoleucine on liver metabolism are associated with higher insulin sensitivity and improved energy balance regulation. This leads to the transformation of white adipose tissue into beige adipose tissue, resulting in increased energy expenditure^42^. Not surprisingly, among the significant pathways involved in the NP group, we found BCAA biosynthesis and degradation, as well as other amino acid biological pathways, such as glutathione metabolism. The tripeptide glutathione, composed of glutamic acid, glycine, and cysteine, plays an essential role in the oxidative metabolism pathway in the liver^43^. Glutathione also participates in the network, controlling the decision between survival, necrosis, and apoptosis, as well as changing the activity of transcription factor and signal transduction molecules^44^. These findings corroborate our previous study^10^, which also identified the effects of prenatal nutrition on oxidative metabolism in the liver.

In a holistic view (omics integration), we identified exclusive biological pathways associated with the prenatal nutritional strategy that did not receive energy-protein supplementation (NP group). Even after the metabolomics-transcriptomics integration, a few of the pathways identified in the previous analyses (such as glycolysis or gluconeogenesis, histidine metabolism, and cholesterol metabolism) continued to be substantially linked to the NP group. This indicates that these biological pathways are thought to be of utmost significance since they are linked to the NP group in at least two out of the three analyses performed. Aminoacyl-tRNA biosynthesis and nitrogen metabolism were also identified in the integrative analysis. They have a strong relationship with amino acid metabolism and translational processes, which were biological pathways discovered in metabolomics and transcriptomics functional enrichment analyses, respectively.

Additionally, biological pathways associated with regulatory and epigenetics mechanisms were highlighted (PPAR signaling pathway, Hippo signaling pathway). Peroxisome proliferators-activated receptors (PPARs) are steroid hormone receptors crucial for lipid metabolism and nutrient sensing. The liver’s most significant tasks are controlling adipogenesis^45^, oxidative metabolism, and fatty acid transport^46^. Many regulators of PPAR and factors regulated by PPAR are involved in epigenetic mechanisms, including non-coding RNAs, enzymes that modify epigenetic marks on histones, and enzymes that add methyl groups to DNA^47^. The hippo-signaling pathway regulates cell growth and organ size^48^. Abnormal activation can alter the liver epithelial cell proliferative state^49^ and the inactivation of the hippo pathway in hepatocytes led to dedifferentiation^50^. Regarding the hub gene and hub metabolite identified in the integrative analysis of NP group, the *SLC6A14*, and the glutamic acid play important roles (amino acid transporter and glutathione metabolism, respectively) linked to the biological processes emphasized in the previous analyses.

### PP group revealed association with several types of biological processes

From the five PP group significant gene modules, the cyan module (*r* = 0.61) and antiquewhite4 module (*r* = -0.54) were identified as the most correlated with the PP treatment. The cyan module hub gene (*ETF1*) is a protein-coding gene essential for the translation termination process. The *ETF1* gene encodes eRF1, a crucial translation termination factor. It was shown that *ETF1* was downregulated in the liver and colon and expressed at a high level in the testis, brain, heart, and kidney^51^. When stop codons (UAA, UAG, or UGA) are present in mRNA, the *ETF11*/eRF1 gene plays a significant role in releasing freshly produced proteins^52^. The hub gene in the antiquewhite4 module, *SIAE* (Sialate O-Acetyl esterase gene), is associated with acetylation process. 9-O-acetylation of sialic acid affects *CD22*, a lectin that binds to sialic acid on B cells and controls B cell activation^53^. The removal of acetyl groups from sialic acid by the enzyme *SIAE* allows *CD22* to operate as intended^54^. In animal studies, protein deficiencies in SIAE and CD22 cause hyperactive B cells and autoimmunity^55^. In general, the biological processes identified in the PP significant modules are associated with regulatory mechanisms and epigenetic factors (mRNA splicing, Via spliceosome (GO:0000398); RNA splicing, via transesterification reactions with bulged adenosine as nucleophile (GO:0000377); regulation of translation (GO:0006417); DNA repair (GO:0006281)), although there are biological processes involved in energy metabolism (Unsaturated Fatty Acid Metabolic Process (GO:0033559); Gluconeogenesis (GO:0006094)), amino acid metabolism (Serine Family Amino Acid Metabolic Process (GO:0009069)) and immune system (Endocytosis (GO:0006897)).

Related to the co-expressed metabolite modules, two of them were significantly correlated with the PP group (red and turquoise). The PC ae C34:2 (Phosphatidylcholine acyl-alkyl C34:2; red module) and PC ae C44:5 (Phosphatidylcholine acyl-alkyl C44:5; turquoise module) were considered the hub metabolites of the PP treatment. Phosphatidylcholines are part of the primary category of lipids known as phospholipids, which are the most abundant lipids found in eukaryotic cells^56,57^. These lipids are essential for the biosynthesis of functioning membranes, and their makeup has a significant impact on the fluidity, permeability, and temperature phase behavior of cell membranes^58^. In the liver, it provides antioxidant protection in the plasma membrane of hepatocytes^59^, assists in the solubilization of cholesterol in bile^60^ and promotes lipoprotein assembly and secretion^61^. No metabolic pathway was functionally enriched in the red module; however, one metabolic pathway (arginine and proline metabolism) was associated with the turquoise module. Arginine is a highly versatile amino acid. Apart from being an essential element in the production of proteins, it also functions as a precursor for polyamines, creatine, and nitric oxide. Glutamate and proline can be converted from arginine to drive cell growth by activating mTORC1 ^62^. Furthermore, some studies identified arginine as an epigenetic regulator^63^ and metabolic modulator of T cells^64^. In addition to being a key substrate for the biosynthesis of arginine, proline is also vital for all animals to have optimal health, optimized collagen production, improved growth performance^65^ and has free radical scavenging potential^66^.

In the integrative analysis of PP group, we investigated the transcriptome-metabolome exclusive biological pathways involved with the prenatal protein-energy supplementation in the final third of pregnancy. Our investigation revealed that a metabolic pathway sustained enrichment even post-integrative analysis (arginine and proline metabolism). This highlights the significance of the arginine and proline metabolism pathway in the context of prenatal energy-protein supplementation during the last trimester of pregnancy in beef cattle. The most significant exclusive pathway found was the TGF-beta signaling pathway. Transforming growth factor-β (TGF-β) represents an evolutionarily conserved family of secreted polypeptide factors that regulate many aspects of physiological embryogenesis, cell growth, differentiation, and cellular homeostasis in animals^67,68^. In animal production, this pathway has already been related to meat composition in pigs^69^, heat stress in beef cattle^70^, muscle fatty acid profile of Nelore cattle^68^ and nutrigenomic studies^71,72^. The glyoxylate and dicarboxylate metabolism is related to gluconeogenesis, ureagenesis^73^ and fatty acid oxidation in the liver^74^. This pathway has been associated with heat stress in steers^75^, fetal growth restriction^76^ and fetal programming in beef cattle^77^. Regarding the hub metabolite identified in the integrative analysis of PP group, arginine plays crucial roles (epigenetic modulator, immune system functions, protein production) and is linked to the biological processes earlier emphasized. The hub gene (*ODC1*) encodes the enzyme necessary for the prompt and effective production of polyamines during liver regeneration^78^. Additionally, *ODC1* is essential for the growth, development, and antioxidant activity of cells^79^. Briefly, the addition of protein and energy in the cows’ diet during the final third of pregnancy (PP group) impacted different biological processes, metabolic pathways, and hub components. These changes are associated with variations in oxidative and energy metabolism and in regulatory and epigenetic functions, all of which play an important role in several critical livestock traits and are essential to animal production performance.

### FP group is significantly associated with immunological mechanisms

The lightcoral and yellow4 gene modules associated with FP group exhibited the strongest correlations (*r* = -0.47 and *r* = 0.46, respectively). The lightcoral hub gene, *PTPRC* (Protein Tyrosine Phosphatase Receptor Type C, also known as *CD45*), is expressed in nearly all hematopoietic cells except mature erythrocytes, playing a vital role in regulating T and B cell antigen receptor-mediated activation. Imbalances in protein tyrosine kinase and phosphatase activity can lead to conditions such as immunodeficiency, autoimmunity, or malignancy^80^. This gene has already been identified as differentially expressed in fetal mouse liver subjected to maternal food restriction^81^. *SLC12A8* (Solute Carrier Family 12 Member 8), the hub gene of yellow4 module, belongs to the solute carrier (SLC) transporters family. It plays a crucial role in regulating the transportation of substances across biological membranes, being essential for maintaining the balance of substances (including inorganic ions, amino acids, lipids, sugars, neurotransmitters, and drugs) within the organism and vital for overall cellular function^82^. Other study associated this gene with maintenance of blood pressure and fluid balance in the body^83^. This gene has also been found to be differentially expressed in the liver of cows 21 days after calving, with varying body condition scores (normal vs. high)^84^. The enhanced processes are tightly linked to the hub genes’ activities, the same as what is seen in the previous treatments. Therefore, we can state that bulls that received protein and energy throughout gestation (FP group) had higher correlations with immunological mechanisms (e.g., T Cell Activation (GO:0042110); T Cell Differentiation (GO:0030217); Positive Regulation of B Cell Activation (GO:0050871); Regulation of Immunoglobulin Production (GO:0002637)). In addition, Negative Regulation of Nucleic Acid-Templated Transcription (GO:1903507) and Ephrin Receptor Signaling Pathway (GO:0048013) are biological pathways involved in cellular regulatory mechanisms. Regulation of cardiac Muscle Cell Differentiation (GO:2000725) was considered a false positive, as it was not related to gene expression in the liver.

### What is the relationship among the prenatal nutrition groups?

The results found in the WGCNA analyses are intricately related to the final results of this study, since the significant modules were used as input for over-representation analyses and for the integration analysis. To explore the interactions among the prenatal nutrition groups, we discussed here the main points to be analyzed.

We found several biological processes and three gene co-expression modules in common among the prenatal groups. However, the correlation signal, magnitude and quantity of the biological pathways were differently related for each group. The palevioletred3 showed a positive correlation with NP group (*r* = 0.47), while the PP group was negativelly correlated with it (*r* = -0.49). The darkgrey module demonstrated the opposite, being the NP group negatively correlated (*r* = -0.67) and the PP group positively correlated (*r* = 0.51). Lastly, the cyan module was shared between the NP group (*r* = -0.5) and FP group (*r* = 0.61). Therefore, the biological processes associated with these shared modules between treatments, despite showing the same enriched pathways, exhibit distinct mechanisms of action, as the correlation signs of gene modules are opposite between treatments. The hub genes such as *EIF4G2*, *GSS*, and *ETF1* were identified in these modules, exhibiting either positive or negative associations depending on the treatment group. *EIF4G2* plays a role in translation and cellular stress responses^31,32^, showed differential associations between the groups, suggesting it may contribute to distinct stress-adaptive mechanisms across prenatal treatments. *GSS* (Glutathione synthetase), crucial for glutathione biosynthesis and cellular detoxification^85^, may have varying effects on oxidative stress management depending on nutritional context, as its expression was also group-dependent. *ETF1*, involved in translation termination^52^, may reflect differences in the regulation of protein synthesis across the groups. These findings highlight that, although the modules are enriched for similar biological pathways, the opposite correlation patterns of these hub genes across treatments point to distinct mechanistic responses.

More than 90% of the enriched biological processes in the shared modules (palevioletred3, darkgrey and cyan) are associated with regulatory and epigenetic mechanisms, which is indicative that the prenatal nutrition can modulate long-term regulatory and epigenetic mechanisms in the liver of Nelore bulls. Furthermore, the most of the significant biological processes found were exclusively correlated with only one group, also indicating possible long-term effects of fetal programming in beef cattle, as discussed in the preceding sections. While further research is needed to understand the specific effects of prenatal nutrition on each biological process, this study strongly contributes to the field of fetal programming in beef cattle and its underlying molecular mechanisms.

## Conclusions

In summary, the hub components and the biological pathways were involved with energy, protein and oxidative metabolism, regulatory and epigenetic processes, and immune function. Based on the frequency, magnitude, correlation signal and exclusive biological pathways in each nutritional treatment group, we can conclude that FP group had a higher correlation with immune mechanisms, while PP and NP groups showed a relationship with regulatory and epigenetics mechanisms. It is worth noting that the shared modules between the groups exhibited opposite correlations, potentially indicating epigenetic changes triggered by prenatal nutrition, along with the exclusive modules correlated within each group. Additionally, the integration of liver transcriptome and metabolome data provided intriguing insights into fetal programming in beef cattle, revealing key biological pathways and hub compounds that were both in line with prior analyses and novel discoveries.

## Electronic supplementary material

Below is the link to the electronic supplementary material.

Supplementary Material 2

Supplementary Material 1

## Data Availability

The transcriptome datasets analysed during the current study are available in the European Nucleotide Archive (ENA) repository (EMBL-EBI), under accession PRJEB75582 [http://www.ebi.ac.uk/ena/browser/view/PRJEB75582]. The metabolomic data analysed during this study are included in this published article [Additional file 2].

## Abbreviations

BCAA: Branched-chain amino acids
BW: Body weight
CCW: Cold carcass weight
CKAP2: cytoskeleton associated protein 2
CP: Crude protein
DMI: Dry matter intake
EIF4G2: Eukaryotic translation initiation factor 4 gamma 2
ETF1: Eukaryotic translation termination factor 1
FIA-MS/MS: Flow injection analysis-tandem mass spectrometry
FP: Full programming
HCW: Hot carcass weight
HPLC: High-performance liquid chromatography
HPLC-MS/MS: Liquid chromatography tandem-mass spectrometry
GSS: Glutathione synthetase
KEGG: Kyoto encyclopedia of genes and genomes
LOD: Limits of detection
LW: Liver weight
MAPA: Ministry of Agriculture, Livestock, and Supply of Brazil
NDF: Neutral detergent fiber
NEFA: Non-esterified fatty acids
NP: Not programmed
ODC1: Ornithine decarboxylase 1
PC ae C34: 2:Phosphatidylcholine acyl-alkyl C34:2
PC ae C44: 5:Phosphatidylcholine acyl-alkyl C44:5
PP: Partial programming
PPARs: Peroxisome proliferators-activated receptors
PTPRC: Protein Tyrosine Phosphatase Receptor Type C
r: correlation
REA: Ribeye area
RIN: RNA integrity number
SFT: Subcutaneous fat thickness
SIAE: Sialate O-Acetyl esterase gene
SLC12A8: Solute Carrier Family 12 Member 8
SLC6A14: Solute carrier family 6 member 14
ST3GAL5: ST3 beta-galactoside alpha-2,3-sialyltransferase 5
TDN: Total digestible nutrients
TGF-β: Transforming growth factor-β
WGCNA: Weighted gene co-expression network analysis
ZNF311: Zinc finger protein 311
