# Supplementary material for: Liver transcriptomics-metabolomics integration reveals biological pathways associated with fetal programming in beef cattle
Source: Sci Rep. 2024 Nov 12;14:27681. doi: 10.1038/s41598-024-78965-4 (PMC11557885; doi:10.1038/s41598-024-78965-4)
Supplement: Supplementary file 2 — Supplementary Material 1 [file 41598_2024_78965_MOESM2_ESM.pdf]

Supplementary material

Table S1. Sequencing data samples with initial filters and multimapping reads, uniquely mapped and unmapped reads rates.

samples	initial_reads	reads_seqyclean	unique_reads	%	multi_mapping_reads	unmapped reads
6781_R1	20876217	18961131	17627729	92.97%	2.96%	4.07%
6781_R2	20876217	18961131				
6796_R1	21309425	19351095	18032506	93.19%	2.94%	3.86%
6796_R2	21309425	19351095				
6797_R1	18545907	16895740	15810376	93.58%	2.94%	3.49%
6797_R2	18545907	16895740				
6808_R1	18543874	16840462	15640834	92.88%	2.91%	4.21%
6808_R2	18543874	16840462				
6816_R1	17965188	16341374	15248030	93.31%	3.03%	3.65%
6816_R2	17965188	16341374				
6821_R1	22594158	20568374	19017014	92.46%	3.02%	4.53%
6821_R2	22594158	20568374				
6822_R1	18769064	17097028	16073556	94.01%	2.97%	3.01%
6822_R2	18769064	17097028				
6876_R1	21856751	19806332	18615402	93.99%	2.87%	3.14%
6876_R2	21856751	19806332				
6979_R1	17220956	15703579	14512131	92.41%	3.02%	4.56%
6979_R2	17220956	15703579				
7045_R1	19383228	17521667	16455068	93.91%	2.45%	3.64%
7045_R2	19383228	17521667				
7063_R1	25881448	23464316	21853881	93.14%	2.92%	3.95%
7063_R2	25881448	23464316				
7072_R1	20754154	18901391	17672424	93.50%	2.95%	3.56%
7072_R2	20754154	18901391				
7074_R1	17626625	16030167	14860689	92.70%	3.08%	4.21%
7074_R2	17626625	16030167				
7075_R1	15626988	14263812	13221182	92.69%	2.91%	4.40%
7075_R2	15626988	14263812				
7085_R1	18547876	16835816	15682028	93.15%	2.92%	3.94%
7085_R2	18547876	16835816				
